# Mortality salience and helping behavior amidst public crisis: cross-sectional evidence during COVID-19

**DOI:** 10.3389/fpubh.2024.1455818

**Published:** 2024-10-08

**Authors:** Qing Xie, Yaqin Yan, Ji Lai, Meiting Wei

**Affiliations:** ^1^Department of Management, Hunan Police Academy, Changsha, China; ^2^Department of Student Affairs, Hunan First Normal University, Changsha, China; ^3^Faculty of Education, Yunnan Normal University, Kunming, China

**Keywords:** mortality salience, COVID-19, helping behavior, death anxiety, death reflection

## Abstract

**Background:**

As a real mortality salience, a public crisis would have a major impact on individual minds, behaviors, and lifestyles. COVID-19 provides us with a stark real-world example to understand these implications. Previous research has revealed that some individuals become more willing to help the infected at the risk of their own lives, while others become more self-centered and indifferent during COVID-19. To explain this paradoxical phenomenon, our study used two rival mediators in the relationship between mortality salience and helping behavior during COVID-19: death anxiety and death reflection.

**Methods:**

A cross-sectional survey was conducted among Chinese college students (N = 684) during the pandemic. We used a parallel mediation model to explore the mediating roles of death anxiety and death reflection in the relationship between mortality salience and helping behavior during COVID-19.

**Results:**

The results of our study indicate two key findings. First, mortality salience is negatively related to helping behavior during COVID-19 via death anxiety. This suggests that individuals with higher levels of mortality salience experienced increased death anxiety, which in turn led to a decrease in helping behavior. Second, mortality salience is positively related to helping behavior during COVID-19 via death reflection. This indicates that individuals with higher levels of mortality salience engaged in deeper reflection on death, which subsequently resulted in an increase in helping behavior.

**Conclusion:**

Our study provides valuable insights into the complex relationship between mortality salience and helping behaviors in the time of public crisis, and can help lead to more positive attitudes toward public crisis events such as COVID-19.

## 1 Introduction

Mortality salience refers to the activation of an individual’s awareness of death (1). In daily life, people are often reminded of their mortality through public crisis events such as widespread infectious diseases, natural disasters, and safety accidents. According to data released by the Ministry of Emergency Management of China, various natural disasters in China have affected 95.444 million people, resulted in 691 deaths or missing persons, and caused direct economic losses of 345.45 billion RMB in 2023 (2). Meanwhile, helping behavior is characterized by the voluntary and proactive efforts of an individual to show care and provide assistance to others (3). As a common type of prosocial behavior, helping behavior is praised and promoted by human society, which plays an important role in combating public crisis and preserving social stability (4). However, helping behavior during public crises also often incurs significant costs and poses threats to the life and safety of the helper. So, what choices do individuals make when faced with mortality salience induced by a public crisis?

The COVID-19 pandemic has provided a real-world context for exploring this question. Some studies have found that helping behavior increased during COVID-19 (5, 6). For example, many people participated in volunteer activities, risking infection to help measure people’s temperatures and deliver necessities to those in quarantine. Meanwhile, faced with the threat of COVID-19, some individuals become more self-centered and indifferent, such as violating epidemic prevention regulations, hoarding products, and reducing their altruistic behavior (7, 8). In order to elucidate this paradox, we conducted a cross-sectional survey in the context of COVID-19, aiming to investigate the underlying mechanisms through which mortality salience influences helping behaviors during public crises. Through this attempt, we could deepen our understanding of the relationship between mortality salience and helping behavior. More importantly, it could help to better promote people’s helping behavior in times of public crisis.

### 1.1 Mortality salience and helping behavior during COVID-19

According to Terror Management Theory (TMT), when individuals are reminded of their mortality, the awareness of death would conflict with the human instinct for survival, leading to anxiety and fear (9). In order to cope with this negative state, individuals would engage in a range of defensive behaviors, including proximal defense and distal defense (10). The former refers to when people have thoughts related to death, they will rationally suppress these thoughts and remove them from awareness; the latter refers to people changing their cognition, emotions, and behavior to enhance self-esteem and maintain the cultural worldview, so that individuals believe that their valuable parts in the world will continue.

What is the relationship between mortality salience and helping behavior? The current evidence points to two distinct conclusions. Some studies have demonstrated that mortality salience could promote helping others (11, 12). Belmi and Pfeffer conducted a longitudinal study in which they manipulated mortality or toothache experiences for the participants (13). Over the following week, participants in the mortality salience condition reported engaging in more actual helping behaviors. Meanwhile, other studies have found that mortality salience could lead to increased indifference, prejudice, and selfishness (14–17). Bassett and Connelly’s research showed that mortality salience can lead to increased hostility toward outgroups (18). This suggests that individuals in the mortality salience condition may be less willing to help others.

COVID-19 could be viewed as a real mortality salience due to its terrible mortality rate, highly contagious nature, and a large number of messages or cues about the epidemic. In the context of the COVID-19 pandemic, there is also a similar contradictory trend in people’s helping behaviors. On one hand, some studies demonstrate that altruism increased during the COVID-19 epidemic (19, 20). For example, Jin and Ryu conducted a manipulation of mortality salience induced by COVID-19, and they found that participants in the mortality salience condition were more willing to help others (21). On the other hand, other studies have shown that during the pandemic, mortality salience led individuals to be less willing to participate in voluntary epidemic prevention activities and to get vaccinated (22, 23).

This contradictory trend suggested that the influence of mortality salience on helping behavior during COVID-19 may involve a complex or even competing psychological mechanism. According to TMT, external mortality cues would evoke internal death awareness, thus leading to various defensive behaviors. According to the contingency model of death awareness, death awareness consists of two basic forms: death anxiety and death reflection (24). We expected death anxiety and death reflection to play competing mediating roles. Death anxiety may lead to self-protective motivation, thereby reducing helping behavior during COVID-19, while death reflection may lead to prosocial motivation, thereby promoting helping behavior during COVID-19.

Therefore, we are not proposing a hypothesis about the relationship between mortality salience and helping behavior during COVID-19 here.

### 1.2 The mediating role of death anxiety

According to TMT, death anxiety could be triggered by mortality salience, including imagining one’s own death, walking past a funeral home, watching death videos, and reading death-related news (18, 25, 26). It has been demonstrated that these manipulations could increase individuals’ state of death anxiety (27, 28). During the outbreak of COVID-19, individuals were exposed to plentiful mortality clues related to death, which also lead to increased death anxiety (29). For example, Hu and colleagues confirmed that mortality salience triggered by COVID-19 was positively related to death anxiety (6).

Meanwhile, based on TMT, death anxiety can lead to self-protection motivation, triggering distal defense mechanisms such as self-esteem seeking and cultural worldview defense. Considering that helping others is a widely advocated social norm, research has found that helping behavior is also an important way to defend against death anxiety (6, 13). However, death anxiety does not promote all types of helping behavior. Proximal defense mechanisms suggest that situational cues inherent in helping behaviors, which serve to remind participants of their own mortality or even pose threats to their lives, can impede the management of death anxiety. This, in turn, may lead to a decrease in their willingness to help others when faced with mortality salience. For example, some studies have found that death anxiety leads to more negative attitudes toward the older adult and the disabled (30, 31). Additionally, Hirschberger and colleagues confirmed that mortality salience leads participants to be less willing to participate in organ donation or assist people with disabilities in wheelchairs (11). Fan and colleagues also found that individuals under mortality salience were less likely to participate in voluntary epidemic prevention activities (22).

During the COVID-19 epidemic, helping those who have been infected means a higher likelihood of becoming infected with COVID-19. A study indicates that during the pandemic, the mortality rate increased by 5.1%, and the life expectancy decreased by 1.6 years (32). Meanwhile, contact with the infected can also easily lead individuals to think about the physicality and vulnerability of the body. Hence, given that helping behavior during COVID-19 can easily elicit individuals’ perception of mortality, those with high death anxiety will be less willing to engage in helping behavior during COVID-19. In addition, some studies also demonstrated death anxiety can contribute to reduced wellbeing (increased uncertainty, depression, and emotional exhaustion), which may also lead to more withdrawal behaviors and less desire to help others (33, 34).

In conclusion, our hypothesis was as follows:

*H1*: Death anxiety mediates the negative relationship between mortality salience and helping behavior during COVID-19.

### 1.3 The mediating role of death reflection

In contrast to ‘death anxiety’, Cozzolino introduced the concept of ‘death reflection’, after summarizing research on TMT, near-death experiences (NDE), and post-traumatic growth (PTG) (35). Individuals in the state of death reflection will place their lives in a larger context, meditate on their meaning and purpose, and consider how others will view them after their death (36).

According to the contingency model of death awareness, mortality salience during COVID-19 may also lead to death reflection. Death anxiety describes the emotional (‘hot’) profile of death awareness based on experiential processing, while death reflection describes the cognitive (‘cool’) profile of death awareness based on cognitive processing. Shao and colleagues confirmed that COVID-19 information exposure was positively related to death reflection (5). Zhong found that death reflection had become the most prevalent form of death awareness during the COVID-19 epidemic (34).

Meanwhile, Grant and Wade Benzoni argue that death reflection is a positive aspect of death awareness, which can enhance an individual’s prosocial motivation (24). Individuals who are in the process of death reflection will become more concerned with the value and meaning of life, yearning to establish connections with others, and engaging in work that is valuable to society (33). Numerous studies have found that death reflection is positively associated with openness to experience gratitude and meaning in life (37–39). Death reflection may contribute to a shift in individual values in the form of a decrease in extrinsic values such as status and money and an increase in intrinsic values such as spirituality and family (35, 40). Death reflection can also motivate individuals to be less greedy, more helpful in the workplace, and more involved in blood donation activities (5, 35, 37). All of these pieces of evidence suggest that death reflection could promote helping behavior during COVID-19, even if these actions might be risky or arouse awareness of death.

In conclusion, our hypothesis was as follows:

*H2*: Death reflection mediates the positive relationship between mortality salience and helping behavior during COVID-19.

### 1.4 The current study

Recent studies have confirmed that mortality salience has an inconsistent effect on helping behavior during COVID-19. Our research aimed to elucidate this phenomenon by examining the mediating roles of two competing factors: death anxiety and death reflection. Additionally, most previous studies have focused on the respective roles of death anxiety and death reflection. Studies testing the joint effect of the two variables simultaneously are still sparse (34, 41). In the current research, we investigated the joint effects of death anxiety and death reflection on individuals’ helping behavior in the context of COVID-19.

**Figure 1:**
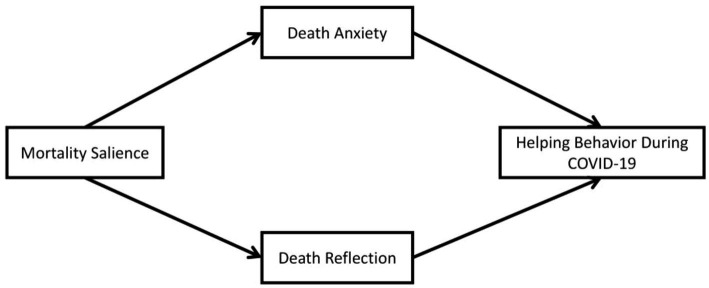
The research model.

To validate our research hypotheses (see Figure 1), we conducted a cross-sectional survey (*N* = 684) with Chinese college students in December 2022 (when the pandemic was still ongoing). This study was conducted according to the guidelines of the Declaration of Helsinki, and approved by the Institutional Review Board of the author’s institution.

## 2 Study

### 2.1 Design and participants

Using a convenient sampling method, we recruited 694 students from universities in China. They all consented to participate in our study and provided their own data via online surveys. We measured their levels of mortality salience, death anxiety, death reflection, and helping behavior during COVID-19. In total, 10 participants were excluded for non-serious answering (failed the attention check or provided patterned responses). Our final sample consisted of 684 respondents (*M_age_* = 21.49 years, SD_age_ = 2.16 years; 67.50% women).

### 2.2 Procedure and measures

First, participants were informed that this was a test about personality traits. Next, participants needed to complete the Mortality Salience Scale, Death Anxiety Scale, and Death Reflection Scale. Then, participants would see a description of a charity project, seeking volunteers helping to fight against COVID-19. They were asked to indicate their willingness to participate. Finally, participants’ age, sex, and subjective family socioeconomic status (SES) were also collected as control variables. Each participant received 10 RMB for their participation.

#### 2.2.1 Mortality salience

We used the Mortality Salience Scale adapted from Shao to measure participants’ mortality salience during COVID-19 (5). The scale consists of 4 items (e.g., ‘During COVID-19, how frequently do you check the death information from the internet’, 1 = never to 5 = frequently). Higher scores indicate a higher degree of mortality salience. The internal consistency coefficient of the scale is 0.83 in our research.

#### 2.2.2 Death anxiety

We used the Death Anxiety Scale developed by Templer (42). The scale consists of 15 items (e.g., ‘I am very much afraid to die’, 1 = strongly disagree to 5 = strongly agree). Higher scores indicate a higher degree of death anxiety. The internal consistency coefficient of the scale is 0.81 in our research.

#### 2.2.3 Death reflection

We used the Death Reflection Scale developed by Yuan (33). The scale consists of 15 items (e.g., ‘When I think about death, I feel like I should do more for the world’, 1 = strongly disagree to 5 = strongly agree). Higher scores indicate a higher degree of death reflection. The internal consistency coefficient of the scale is 0.84 in our research.

#### 2.2.4 Helping behavior during COVID-19

Referring to Fan (22), participants were asked to read a short article introducing the Chinese Youth Volunteer Association, and were informed that the association was launching a project to combat COVID-19. The project aims to recruit volunteers to participate in the fight against the pandemic in high-risk areas of China. Volunteers’ main duty is to aid quarantined individuals in overcoming daily living difficulties. The association will provide basic living guarantees for the volunteers, but there is a certain risk of infection in the voluntary service. Finally, the participants were required to express their willingness to participate (1 = very unwilling to 5 = very willing).

## 3 Results

### 3.1 Common method Bias test

The common method bias was investigated using Harman’s single-factor test. The results revealed that without rotation, there were nine factors with eigenvalues greater than 1. The first factor explained 16.08% of the variance, which is below the critical standard of 40% (43). This indicates that the common method bias in this study is not serious.

### 3.2 Descriptive statistics and correlation analysis

Table 1 displays the results of descriptive statistics and correlations among our focus variables. Pearson’s correlation coefficients of our focus variables showed that mortality salience was significantly correlated with death anxiety (*r* = 0.17, *p* < 0.01), death reflection (*r* = 0.29, *p* < 0.01), and helping behavior during COVID-19 (*r* = 0.17, *p* < 0.01); death anxiety was significantly correlated with helping behavior during COVID-19 (*r* = −0.15, *p* < 0.01); death reflection was significantly correlated with helping behavior during COVID-19 (*r* = 0.32, *p* < 0.01).

**Table 1:** Descriptive statistics and correlations among variables in our study.

Variables	*M*	SD	1	2	3	4	5	6	7
1. Sex	0.68	0.47	1						
2. Age	21.49	2.16	−0.10	1					
3. SES	4.32	1.72	−0.06	0.01	1				
4. Mortality salience	3.52	0.58	0.03	0.05	0.01	1			
5. Death anxiety	3.34	0.60	0.14^*^	−0.08	−0.03	0.17^**^	1		
6. Death reflection	3.73	0.61	−0.13^*^	0.04	0.00	**0.29** ^**^	0.09	1	
7. Helping behavior during COVID-19	3.16	1.07	−0.14^*^	0.07	0.04	**0.17** ^**^	**−0.15** ^**^	**0.32** ^**^	1

*N* = 684. Sex (men = 0, women = 1). ^*^p < 0.05; ^**^p < 0.01; ^***^p < 0.001 (two-tailed). Correlation coefficients between the focus variables are indicated by the bold values.

### 3.3 Mediation analysis

Utilizing the non-parametric percentile bootstrap method for mediation effect testing proposed by Hayes, we examined the mediation effects of death anxiety and death reflection by employing model 4 of PROCESS macro of SPSS 23 (44).

As shown in Tables 2, 3, mortality salience was positively related to death anxiety (*β* = 0.17, *t* = 4.49, *p* < 0.001). Death anxiety was negatively related to helping behavior during COVID-19 (*β* = −0.18, *t* = 5.00, *p* < 0.001). Importantly, the indirect effect of mortality salience on helping behavior during COVID-19 via death anxiety was significant (*Index* = −0.03, *SE* = 0.01, 95% CI [−0.06, −0.01]), supporting Hypothesis 1.

**Table 2:** Mediation analytic results in the study.

Independent variables	Dependent variables					
	Death anxiety		Death reflection		Helping behavior during COVID-19	
	*β*	*t*	*β*	*t*	*β*	*t*
Mortality salience	0.17	4.49^***^	0.29	7.94^***^	0.12	3.20^*^
Death anxiety					−0.18	−5.00^***^
Death reflection					0.29	7.71^***^
Sex	0.12	3.24^*^	−0.14	−3.70^*^	−0.08	−2.14
Age	−0.07	−1.90	0.01	0.39	0.03	0.79
SES	−0.02	−0.49	−0.01	−0.32	0.03	0.87
*R*	0.28		0.32		0.39	
*R* ^2^	0.08		0.10		0.15	
*F*	11.07^***^		19.01^***^		20.38^***^	

*N* = 684. ^*^p < 0.05; ^**^p < 0.01; ^***^p < 0.001 (two-tailed). All variables in the model are standardized before being incorporated into the regression equation.

**Table 3:** The mediating role of death anxiety and death reflection in our study.

	Index	SE	95% CI
Mortality salience → Death anxiety → Helping behavior during COVID-19	−0.03	0.01	[−0.06, −0.01]
Mortality salience → Death reflection → Helping behavior during COVID-19	0.08	0.02	[0.04, 0.13]
Direct effect	0.12	0.05	[0.01, 0.22]
Total effect	0.17	0.05	[0.07, 0.28]

Mortality salience was positively related to death reflection (*β* = 0.29, *t* = 7.94, *p* < 0.001). Death reflection was positively related to helping behavior during COVID-19 (*β* = 0.29, *t* = 7.71, *p* < 0.001). Importantly, the indirect effect of mortality salience on helping behavior during COVID-19 via death reflection was significant [*Index* = 0.08, *SE* = 0.05, 95% CI (0.04, 0.13)], supporting Hypothesis 2.

In addition, this study further conducted supplementary analyses to examine the interaction between death anxiety and death reflection on helping behavior during COVID-19. We included the product term of death anxiety scores and death reflection scores in the regression equation, with the other independent variables comprising mortality salience, death anxiety, and death reflection. The dependent variable was helping behavior. The results showed that the interaction between death anxiety and death reflection on helping behavior was not significant (*β* = −0.01, *t* = −0.33, *p* = 0.75).

## 4 General discussion

In conclusion, the current research has explored the dual-channel model of mortality salience influencing helping behavior under the background of the COVID-19 epidemic. In our model, death anxiety and death reflection play opposing mediating roles. This competitive mechanism may explain the double-edged sword effect of mortality salience on helping behavior during COVID-19.

First, our studies found that mortality salience induced by COVID-19 could lead to both death anxiety and death reflection. Based on TMT, external death cues cause awareness of one’s own mortality, leading to a series of defense behaviors (25, 26). Grant and Wade-Benzoni proposed the contingency model of death awareness, further dividing this death awareness into death anxiety and death reflection. Death anxiety engages the hot experiential processing system, while death reflection engages the cool cognitive processing system (24). Consistently, recent studies have shown that mortality salience induced by COVID-19 indeed triggers both people’s death anxiety and death reflection (5, 6, 34). This conclusion is also confirmed in our research.

Second, and more importantly, we found that death anxiety and death reflection played competing mediating roles in the relationship between mortality salience and helping behavior during COVID-19. Most previous studies have explored the separate roles of death anxiety and death reflection. For example, Shao and colleagues found that death anxiety led to more work withdrawal and death reflection led to more helping behavior in the workplace (5). In our study, we explored the joint effects of death anxiety and death reflection on helping behavior during COVID-19. According to TMT, when death anxiety is activated, individuals will develop self-protective motives, resulting in proximal defenses such as avoiding death cues, or distal defenses such as pursuing self-esteem or validating cultural worldviews (45). Previous research has shown that mortality salience can promote helping behavior, which can enhance self-esteem and defend cultural worldviews (12, 13). However, based on the Self-Protective Altruist proposed by Hirschberger, when helping behaviors involve mortality cues (such as organ donation, helping the disabled or the older adult), individuals tend to resort to proximal defense mechanisms, thereby reducing their willingness to engage in helping behaviors (11, 30, 31). During the pandemic, helping others may evoke a sense of mortality or even threaten the lives of the helpers, leading to avoidance by those with high death anxiety. Conversely, studies on death reflection suggest that during COVID-19, individuals may also experience heightened death reflection, which motivates them to be more inclined to contribute to others (24, 36). This prosocial motivation encourages individuals to be more willing to engage in helping behaviors, even when such actions might stir awareness of death. In line with this, previous research has indicated that death reflection can enhance individuals’ readiness to donate blood and support colleagues during COVID-19 (5, 37).

Our dual channel model could explain the double-edged effect of mortality salience on helping behavior during COVID-19. When death anxiety becomes the main death awareness, mortality salience negatively influences helping behavior during COVID-19. When individuals focus more on death reflection, mortality salience positively influences helping behavior during COVID-19. Two studies conducted during the pandemic somewhat support our viewpoint. Zhong and his colleagues conducted a latent profile analysis on the impact of death awareness (34). The results revealed that the calm reflectors (low death anxiety + high death reflection) exhibited more prosocial behaviors than the disengaged (low death anxiety + low death reflection). A longitudinal survey by Jacobsen and Beehr on corporate employees indicated that during the pandemic, employees’ death anxiety negatively predicted prosocial motivation, while death reflection positively predicted prosocial motivation (46).

It should be noted that our research findings might also be explained by other psychological mechanisms. TMT itself also acknowledges the role of mortality salience in facilitating risky behavior and prosocial behavior (26, 47, 48). This suggests that engaging in prosocial behaviors in the context of mortality can also help manage people’s anxiety and fear of death, as long as such behaviors serve to enhance self-esteem and validate cultural worldviews. Routledge and Arndt demonstrated that mortality salience led individuals to be more willing to sacrifice their lives for their country (49). Therefore, it could be that the people who tended to show death anxiety and low willingness to help others during COVID-19 were those with limited terror management resources. They were unable to obtain positive outcomes from helping behaviors to reduce their death anxiety. Meanwhile, those who seemed to be high in death reflection and helping were people with worldviews and bases of self-worth that protected them from death anxiety and guided them toward bolstering their values and self-worth by being willing to help others.

Another interpretation that warrants attention involves our approach to measuring mortality salience. In the classic experimental paradigm of mortality salience, researchers typically elicit distal defense mechanisms through delay tasks (50). However, our study measures the level of mortality salience using a questionnaire. This measure cannot distinguish how much death concerns were in conscious attention and thus activated proximal defense, and how much they were lingering outside of focal attention but accessible and thus activated distal defenses. Mortality salience at the conscious attention might lead to death anxiety and reduce helping behavior during COVID-19, while mortality salience outside of focal attention might lead to death reflection and promote helping behavior during COVID-19.

Additionally, the manner in which death reflection was measured (e.g., ‘When I think about death, I feel like I should do more for the world’) might inherently reflect the degree to which individuals are inclined to help others. This could lead to a positive correlation between death reflection and helping behavior.

## 5 Practical implication

The results of our research have some practical implications. When public crisis events such as COVID-19 occur, fear and anxiety are inevitable. However, our findings confirm that there is still positive energy in the awareness of death. On one hand, we can take certain measures to guide people to manage death anxiety through prosocial behaviors instead of avoidance. The promotion of prosocial deeds and role models could be used to encourage people to associate prosocial behaviors with self-worth and life meaning. We could also organize appropriate forms of helping activities to provide a sense of transcendence beyond death during public crises. For instance, Dunn and colleagues found that mortality salience increased individuals’ willingness to donate signed (self-connection) books, but it had no impact on the donation of unsigned books. Those participants who donated signed books under death reminders reported a higher sense of transcendence (51).

On the other hand, by enhancing individuals’ death reflection, we can also encourage more people to help others during public crises. Death education can be popularized to guide people in looking at and accepting death in a more introspective way. Some propaganda and cultural activities that advocate for death reflection are also feasible approaches. In conclusion, by incorporating helping behavior into an important part of meaning in life, we could reduce the negative impact of death awareness and face various crisis events with a more optimistic outlook.

## 6 Limitations and future direction

Above all, cultural background is an influential factor worth further exploration. The theoretical framework of this report is of Western origin, but the research is conducted in Chinese culture. The Chinese scholars Huang and Hu have proposed a theory of sorrow management, offering a different perspective on death: death is not only the destruction of self but also the separation from the world (especially loved ones); the former triggers fear, and the latter triggers sadness (52). Moreover, Huang and Hu’s findings suggest that sadness was more prevalent than fear among Chinese people, and sadness may be a key mediator of prosocial behaviors that are more likely to be facilitated by death reflection than death anxiety. Subsequent research can further explore the cultural differences in individuals’ attitudes toward death and their emotional reactions.

As a cross-sectional study, our research does not allow for the validation of causal relationships between variables. Subsequent research could further validate causal relationships through longitudinal studies or experiments.

In addition, the current research explored two mediating pathways of death anxiety and death reflection. However, our model did not answer when or under what conditions, mortality salience is more inclined to generate death anxiety or more inclined to generate death reflection. In future, the experimental method can be used to further explore different boundary conditions of the two pathways by manipulating different conditions, such as the attributes of death cues or personal characteristics (such as age, time orientation, or value orientation).

At the same time, considering that the research findings also allow for other possible explanations, subsequent studies could conduct some sophisticated experiments to differentiate and validate these various explanatory frameworks.

Finally, some studies have found that in addition to death anxiety and death reflection, mortality salience may also increase uncertainty or reduce personal control (53, 54). Follow-up studies could examine the relationship between these variables to further clarify the true impact of mortality salience on individuals.

## 7 Conclusion

1. Mortality salience is negatively related to helping behavior during COVID-19 via death anxiety;
2. Mortality salience is positively related to helping behavior during COVID-19 via death reflection.

## Data Availability

The raw data supporting the conclusions of this article will be made available by the authors, without undue reservation.
